# Neuroprotective effects of *Hericium erinaceus* (Bull.: Fr.) Pers. against high-dose corticosterone-induced oxidative stress in PC-12 cells

**DOI:** 10.1186/s12906-020-03132-x

**Published:** 2020-11-11

**Authors:** Sze Yuen Lew, Siew Huah Lim, Lee Wei Lim, Kah Hui Wong

**Affiliations:** 1 Department of Anatomy, Faculty of Medicine, University of Malaya, 50603 Kuala Lumpur, Malaysia; 2 Department of Chemistry, Faculty of Science, University of Malaya, 50603 Kuala Lumpur, Malaysia; 3 Neuromodulation Laboratory, School of Biomedical Sciences, Li Ka Shing Faculty of Medicine, The University of Hong Kong, 21 Sassoon Road, Pokfulam, Hong Kong Special Administrative Region, China, China

**Keywords:** *Hericium erinaceus*, Oxidative stress, Depression mimicking, Endogenous antioxidant, Mitochondrial function

## Abstract

**Background:**

*Hericium erinaceus* is a culinary and medicinal mushroom in Traditional Chinese Medicines. It has numerous pharmacological effects including immunomodulatory, anti-tumour, anti-microbial, anti-aging and stimulation of nerve growth factor (NGF) synthesis, but little is known about its potential role in negating the detrimental effects of oxidative stress in depression. The present study investigated the neuroprotective effects of *H. erinaceus* standardised aqueous extract (HESAE) against high-dose corticosterone-induced oxidative stress in rat pheochromocytoma (PC-12) cells, a cellular model mimicking depression.

**Methods:**

PC-12 cells was pre-treated with HESAE for 48 h followed by 400 μM corticosterone for 24 h to induce oxidative stress. Cells in complete medium without any treatment or pre-treated with 3.125 μg/mL desipramine served as the negative and positive controls, respectively. The cell viability, lactate dehydrogenase (LDH) release, endogenous antioxidant enzyme activities, aconitase activity, mitochondrial membrane potentials (MMPs), intracellular reactive oxygen species (ROS) levels and number of apoptotic nuclei were quantified. In addition, HESAE ethanol extract was separated into fractions by chromatographic methods prior to spectroscopic analysis.

**Results:**

We observed that PC-12 cells treated with high-dose corticosterone at 400 μM had decreased cell viability, reduced endogenous antioxidant enzyme activities, disrupted mitochondrial function, and increased oxidative stress and apoptosis. However, pre-treatment with HESAE ranging from 0.25 to 1 mg/mL had increased cell viability, decreased LDH release, enhanced endogenous antioxidant enzyme activities, restored MMP, attenuated intracellular ROS and protected from ROS-mediated apoptosis. The neuroprotective effects could be attributed to significant amounts of adenosine and herierin III isolated from HESAE.

**Conclusions:**

HESAE demonstrated neuroprotective effects against high-dose corticosterone-induced oxidative stress in an in vitro model mimicking depression. HESAE could be a potential dietary supplement to treat depression.

**Supplementary Information:**

The online version contains supplementary material available at 10.1186/s12906-020-03132-x.

## Background

According to the World Health Organization, more than 300 million people or 4.4% of the world’s population suffer from depression, representing a substantial global health problem. Depression can greatly impact one’s daily life and can lead to suicidal thoughts [1]. In Malaysia, the overall prevalence of depression ranged from 8 to 12% [2], but it is seldom prioritised by patients or clinicians. Currently, the combination of pharmacological and non-pharmacological (including self-care or psychotherapeutic approaches) treatments for depression have only moderate to short-term efficacy. Selective serotonin reuptake inhibitors (SSRIs) are first-line antidepressants commonly used in clinical practice, however, they can have poor tolerability, delayed onset of therapeutic effects and limited efficacy in patients with milder depression [3]. Furthermore, around 50% of patients experience recurrent depression within 6 months after discontinuation of treatments [4]. Therefore, it is important to develop and establish Traditional Chinese Medicines that can mimic the desirable antidepressants effects with minimal adverse consequences.

*Hericium erinaceus* (Bull.: Fr.) Pers. is a medicinal mushroom belonging to the division of Basidiomycota (Aphyllophoromycetideae, Hydnaceae). It grows on dying or dead oak, beech, maple, sycamore, walnut and other broadleaf trees. This mushroom, commonly known as Lion’s Mane, Monkey’s Head (Houtou in Chinese), Hedgehog Fungus, Satyr’s Beard, Pom Pom Blanc, Igelstachelbart and Yamabushitake is native to Europe, North America and Asia. *Hericium erinaceus* has been well-known for over a thousand years in China and other Oriental countries [5]. Its pharmacological effects have been scientifically examined in the past 35 years. Studies involving human subjects at the Third People’s Hospital of Shanghai confirmed its effectiveness against ulcers, inflammations and tumours of digestive tract, and extended the life expectancy of cancer patients [6].

**Fig. 1:**
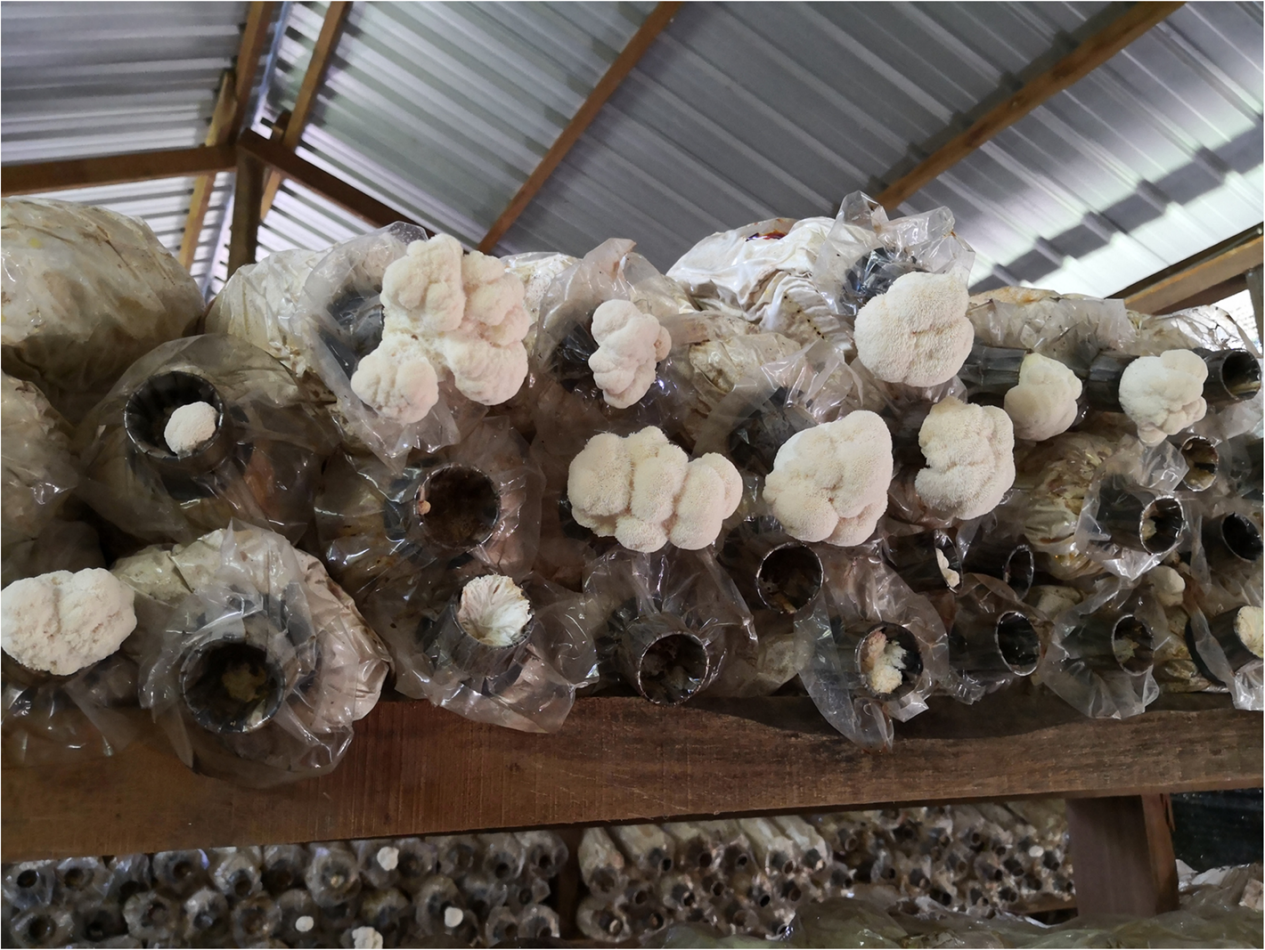
*Hericium erinaceus* mushrooms cultivated in Malaysia. The image was captured at mushroom cultivation farm of Ganofarm R&D Private Limited with permission from Ganofarm R&D Private Limited

Commercial cultivation of *H. erinaceus* began in Malaysia two decades ago (Fig. 1) by mainly using abundantly available waste lignocellulosic materials and rubber-wood sawdust. The isolation and characterisation of diterpenoid derivatives, namely hericenones from the fruiting bodies and erinacines from the mycelium [7], have prompted research into using this mushroom as a functional food.

The pathogenesis of depression remains poorly understood. A generally presumed mechanism of the pathogenesis of depression is that the neuroendocrine hypothalamic-pituitary-adrenal (HPA) axis becomes hyperactive. Normally, the HPA axis is activated in response to stressors and ensures an organism responds appropriately to ever-changing environmental challenges, which is essential for survival. Blood glucocorticoid levels are sensitively controlled by the HPA axis via negative feedback regulation. An elevation in blood glucocorticoids in response to chronic stress or depression can disrupt HPA axis function. Chronic elevation of cortisol secretions in some depressive subtypes is thought to be responsible for cognitive deficits [8], premature osteoporosis [9], ageing [10] and other medical morbidities. Moreover, persistently high levels of blood glucocorticoids can lead to the mobilisation of energy stores (liver, fat and muscle), inflammation, neuronal death and even exacerbate depression [11]. Prolonged elevation of blood corticosterone has been reported to induce depressive-like behaviours in mice [12] and damage to the hippocampal neurons [13].

Studies have demonstrated that glucocorticoids regulate mitochondrial function associated with neuroprotection [14], ROS generation and immune responses [15]. Chronic exposure to glucocorticoids was shown to enhance generation of ROS primarily triggered by damaged mitochondria, diminished cellular adenosine triphosphate (ATP) levels and disrupted antioxidant systems. Furthermore, excessive ROS formation induces oxidative stress, leading to cellular damage and subsequent cell death [16]. Indeed, oxidative stress in and around mitochondria has been postulated to underlie the pathogenesis of mental disorders [17].

Thus, antioxidant defence system comprising of endogenous and exogenous antioxidants are required to combat oxidative stress for optimal cellular functioning and maintain redox homeostasis in living organisms [18]. Exogenous antioxidants can be easily obtained through natural resources including medicinal mushrooms [19]. Typical primary antioxidants include phenolic compounds, phenolic acids and their derivatives, flavonoids, tocopherols, phospholipids, amino acids, phytic acid, ascorbic acid, sterols and pigments. Phenolic compounds act as free radical terminators and able to upregulate the activity of endogenous antioxidant enzymes and therefore indirectly attenuate oxidative stress [20]. The potent antioxidant properties of *H. erinaceus* have been attributed to its remarkably high phenolic acid and flavonoid contents [19, 21]. Administration of *H. erinaceus* appears to have anti-inflammatory properties that can counteract the detrimental effects of free radicals.

Accumulating evidence shows that neuroprotective [22, 23] and neuroregenerative properties [24] of fruiting bodies and mycelium of *H. erinaceus* has led to increasing interest in *H. erinaceus* as a potential therapeutic target for depression [25, 26]. The present study aimed to examine several key questions pertaining to neuroprotective role of *H. erinaceus* in association with cellular and mitochondrial consequences of oxidative stress. We evaluated the neuroprotective effects of *H. erinaceus* standardised aqueous extract (HESAE) in a depression mimicking condition through the establishment of high-dose corticosterone-induced oxidative stress model in PC-12 cells, and analysed the major chemical constituents in the extract. The PC-12 cells possess high levels of glucocorticoid receptors that mediate the rapid response to high-dose corticosterone, which has been widely used as an in vitro experimental model to study depression [27].

## Methods

### Mushroom sample

*Hericium erinaceus* standardised aqueous extract (HESAE; NevGro®; batch No. 7H2308X) was obtained from Ganofarm R&D Private Limited, Tanjung Sepat, Selangor, Malaysia. The extract was prepared by boiling the fresh fruiting bodies with distilled water [1:10 (w/v)] for 7 h and spray-dried with maltodextrin. The trademark was registered with Intellectual Property Corporation of Malaysia (No. 2018001586) and classified as a dietary supplement for human. HESAE was dissolved in Nutrient Mixture F-12 K Ham medium (F-12 K medium; Sigma-Aldrich, St. Louis, MO, USA) and filter-sterilised through a 0.2 μm nylon membrane filter before use.

### PC-12 cell culture

The PC-12 adherent cells (PC-12 cells) were a kind gift from Associate Professor Dr. William Lim Kiong Seng, Universiti Malaysia Sarawak, Malaysia. The cells were cultured in F-12 K medium (Sigma-Aldrich, St. Louis, MO, USA) supplemented with 1% (v/v) penicillin-streptomycin, 15% (v/v) horse serum and 2.5% (v/v) fetal bovine serum at 37 ± 2 °C in a 5% CO_2_-humidified incubator. Cells in F-12 K medium without any treatment or pre-treated with 3.125 μg/mL desipramine (Sigma-Aldrich, St. Louis, MO, USA) served as the negative and positive controls, respectively.

### 3-(4,5-dimethylthiazol-2-yl)-2,5-diphenyltetrazolium bromide (MTT) viability assay

The PC-12 cells were seeded onto a 96-well plate at a density of 1 × 10^5^ cells per well and incubated for 24 h at 37 ± 2 °C in a 5% CO_2_-humidified incubator. The cells were then incubated in F-12 K medium containing various concentrations of corticosterone (EMD Millipore Corporation, Temecula, CA, USA) for 24 h (Experiment I) or HESAE for 48 h (Experiment II). Neuroprotective effects of HESAE was determined by pre-incubating the cells with a selected range of concentrations determined from Experiment II for 48 h followed by high-dose corticosterone determined from Experiment I for another 24 h. After incubation, MTT solution at 0.5 mg/mL was added into each well and further incubated for 4 h at 37 ± 2 °C in a 5% CO_2_-humidified incubator. The medium was then removed and dimethyl sulfoxide (DMSO) was added into each well. Absorbance was measured at 570 nm with 630 nm as background absorbance using a multimode microplate reader (SpectraMax M3; Molecular Devices, San Jose, CA, USA). The cell viability was expressed as a percentage of the negative control level. A high-dose corticosterone determined based on Experiment I (400 μM) was selected for subsequent assays of oxidative stress.

### Lactate dehydrogenase (LDH) assay

The PC-12 cells were seeded onto a 96-well plate at a density of 1 × 10^5^ cells per well and incubated for 24 h at 37 ± 2 °C in a 5% CO_2_-humidified incubator. The cells were then incubated with HESAE or desipramine for 48 h followed by 400 μM corticosterone for 24 h. The 96-well plate was then centrifuged at 250 x *g* for 5 min at room temperature to obtain the supernatant. The LDH release was determined using a LDH cytotoxicity detection kit (Roche, Mannheim, Germany) according to the manufacturer’s protocol. Absorbance was measured at 492 nm with 690 nm as background absorbance using a multimode microplate reader (SpectraMax M3; Molecular Devices, San Jose, CA, USA). The cytotoxicity was expressed as the percentage of the negative control level.

### Superoxide dismutase (SOD) assay

The PC-12 cells were seeded onto a 6-well plate at a density of 5 × 10^5^ cells per well and incubated for 24 h at 37 ± 2 °C in a 5% CO_2_-humidified incubator. The cells were then incubated with HESAE or desipramine for 48 h followed by 400 μM corticosterone for 24 h. Cells were scrapped and collected by centrifugation at 2000 x *g* for 10 min at 4 °C. The pellet was homogenised in 4-(2-hydroxyethyl)-1-piperazineethanesulfonic acid (HEPES) buffer and centrifuged at 1500 x *g* for 5 min at 4 °C to obtain the supernatant. The SOD activity was determined using a SOD assay kit (Cayman Chemical, Michigan, USA) according to the manufacturer’s protocol. Absorbance was measured at 450 nm using a multimode microplate reader (SpectraMax M3; Molecular Devices, San Jose, CA, USA). The activity was expressed as a percentage of the negative control level.

### Catalase (CAT) assay

The PC-12 cells were seeded onto a 6-well plate at a density of 5 × 10^5^ cells per well and incubated for 24 h at 37 ± 2 °C in a 5% CO_2_-humidified incubator. The cells were then incubated with HESAE or desipramine for 48 h followed by 400 μM corticosterone for 24 h. Cells were scrapped and collected by centrifugation at 2000 x *g* for 10 min at 4 °C. The pellet was homogenised in HEPES buffer and centrifuged at 10,000 x *g* for 15 min at 4 °C to obtain the supernatant. The CAT activity was determined using a CAT assay kit (Cayman Chemical, Michigan, USA) according to the manufacturer’s protocol. Absorbance was measured at 540 nm using a multimode microplate reader (SpectraMax M3; Molecular Devices, San Jose, CA, USA). The activity was expressed as a percentage of the negative control level.

### Glutathione peroxidase (GPx) assay

The PC-12 cells were seeded onto a 6-well plate at a density of 5 × 10^5^ cells per well and incubated for 24 h at 37 ± 2 °C in a 5% CO_2_-humidified incubator. The cells were then incubated with HESAE or desipramine for 48 h followed by 400 μM corticosterone for 24 h. Cells were scrapped and collected by centrifugation at 1500 x *g* for 10 min at 4 °C. The pellet was homogenised in phosphate-buffered saline (PBS) and centrifuged at 10,000 x *g* for 10 min at 4 °C to obtain the supernatant. The GPx activity was determined using a GPx assay kit (Elabscience, Wuhan, Hubei, China) according to the manufacturer’s protocol. Absorbance was measured at 412 nm using a multimode microplate reader (SpectraMax M3; Molecular Devices, San Jose, CA, USA). The activity was expressed as a percentage of the negative control level.

### Aconitase assay

The PC-12 cells were seeded onto a 6-well plate at a density of 5 × 10^5^ cells per well and incubated for 24 h at 37 ± 2 °C in a 5% CO_2_-humidified incubator. The cells were then incubated with HESAE or desipramine for 48 h followed by 400 μM corticosterone for 24 h. Cells were homogenised in an assay buffer and centrifuged at 800 x *g* for 10 min at 4 °C to obtain the supernatant. The aconitase activity was determined using an aconitase assay kit (Sigma-Aldrich, St. Louis, MO, USA) according to the manufacturer’s protocol. Absorbance was measured at 450 nm using a multimode microplate reader (SpectraMax M3; Molecular Devices, San Jose, CA, USA). The activity was expressed as a percentage of the negative control level.

### Mitochondrial membrane potential (MMP) assay

The PC-12 cells were seeded onto a 96-well black plate at a density of 1 × 10^5^ cells per well and incubated for 24 h at 37 ± 2 °C in a 5% CO_2_-humidified incubator. The cells were then incubated with HESAE or desipramine for 48 h followed by 400 μM corticosterone for 24 h. Cells were stained according to the manufacturer’s protocol of MMP kit (Sigma-Aldrich, St. Louis, MO, USA). Fluorescence intensities were measured using a multimode microplate reader (SpectraMax M3; Molecular Devices, San Jose, CA, USA) at 540 and 590 nm (red fluorescence), and 490 and 525 nm (green fluorescence) as the excitation and emission wavelengths, respectively. The ratio of red to green fluorescence intensity was expressed as a percentage of the negative control level.

### Intracellular reactive oxygen species (ROS) assay

The PC-12 cells were seeded onto a 96-well black plate at a density of 1 × 10^5^ cells per well and incubated for 24 h at 37 ± 2 °C in a 5% CO_2_-humidified incubator. The cells were then incubated with HESAE or desipramine for 48 h followed by 400 μM corticosterone for 24 h. Then, the medium was removed and the cells were incubated with 10 μM 2′,7′-dichlorofluorescin diacetate (DCFH-DA) (Sigma-Aldrich, St. Louis, MO, USA) for 30 min at 37 ± 2 °C in a 5% CO_2_-humidified incubator. Cells were washed twice with PBS and visualised using the Nikon Eclipse Ti-E inverted microscope with Intensilight C-HGFIE Precentered Fiber Illuminator (Nikon Corporation, Tokyo, Japan). Bright-green fluorescence indicates the oxidation of 2′,7′-dichlorofluorescin (DCFH) to dichlorofluorescin (DCF) in the presence of ROS. The fluorescence intensity was measured using a multimode microplate reader (SpectraMax M3; Molecular Devices, San Jose, CA, USA) at 504 and 538 nm as the excitation and emission wavelengths, respectively. The fluorescence intensity was expressed as a percentage of the negative control level.

### Hoechst 33258 staining of apoptotic nuclei

The PC-12 cells were seeded onto a 96-well black plate at a density of 1 × 10^5^ cells per well and incubated for 24 h at 37 ± 2 °C in a 5% CO_2_-humidified incubator. The cells were then incubated with HESAE or desipramine for 48 h followed by 400 μM corticosterone for 24 h. Then, the medium was removed and the cells were fixed with 4% paraformaldehyde for 10 min at room temperature, followed by incubation with 5 μg/mL Hoechst 33258 (Invitrogen, Carlsbad, CA, USA) for 10 min at 37 ± 2 °C in a 5% CO_2_-humidified incubator. Cells were washed twice with PBS and visualised using the Nikon Eclipse Ti-E inverted microscope with Intensilight C-HGFIE Precentered Fiber Illuminator (Nikon Corporation, Tokyo, Japan) at 460 nm. The number of apoptotic cells in five random non-overlapping fields was quantified by ImageJ [28]. The fluorescence intensity was expressed as a percentage of the negative control level.

### Extraction, isolation and identification of the major compounds of HESAE

The HESAE was macerated and extracted with 95% ethanol. The ethanol extract was then concentrated in vacuo and subjected to two different isolation methods based on size exclusion and polarity. In the size exclusion-based method, ethanol extract was fractionated using gel permeation chromatography [Sephadex LH-20, GE Healthcare, Uppsala, Sweden; methanol (MeOH)] into 10 fractions for which a major compound was derived from the 8th fraction. In the polarity-based method, ethanol extract was partitioned with water and n-butanol into two solvent fractions. The n-butanol fraction was then fractionated using preparative radial chromatography (Silica gel 60 PF_254_, Merck, Darmstadt, Germany). Solvent systems used for preparative radial chromatography were chloroform (CHCl_3_) and 1–15% MeOH−CHCl_3_. The procedure was repeated for three runs to give 10 to 13 fractions. Another major compound was obtained from the 6th fraction of first run, and 5th fraction from second and third runs. The two major compounds were analysed using spectroscopic methods: ^1^H and ^13^C nuclear magnetic resonance (NMR) spectra were recorded in MeOH-*d*_4_ on FT-NMR Avance III 600 MHz (Bruker, Massachusetts, USA), high-resolution electrospray ionisation mass spectrometry (HRESIMS) were obtained on Agilent 6530 Q-TOF mass spectrometer (Agilent Technologies, California, USA), Ultraviolet (UV) spectra were obtained on Shimadzu UV-2600 spectrophotometer (Shimadzu, Kyoto, Japan) and Infrared (IR) spectra were recorded on Spectrum 400 FT-IR/FT-FIR spectrophotometer (PerkinElmer, Massachusetts, USA) [29].

### Statistical analysis

All analyses were performed in Statistical Package for the Social Science (SPSS) 22.0 and data were presented as mean ± standard deviation (SD). Shapiro-Wilk test was employed to evaluate the normality of the data. Normally distributed data were examined with Levene’s test to evaluate the homogeneity of variances between groups. All groups with equal variances assumed were evaluated by one-way analysis of variance (ANOVA) to examine differences between groups, followed by Tukey’s HSD (honestly significant difference) post hoc test. All groups with equal variances not assumed were evaluated by ANOVA, followed by Games-Howell multiple comparison post hoc test. The statistical difference of *p* < 0.05 was considered significant.

## Results

### Effect of corticosterone on the viability of PC-12 cells

**Fig. 2:**
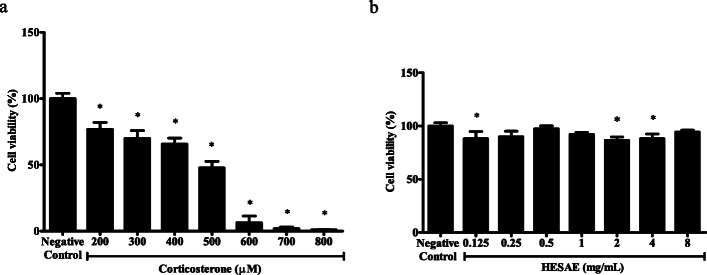
Effect of corticosterone and HESAE on the viability of PC-12 cells. Cell viability was evaluated by MTT assay following incubation with various concentrations of (**a**) corticosterone (200 to 800 μM) for 24 h and (**b**) HESAE (0.125 to 8 mg/mL) for 48 h, respectively. Data are expressed as mean ± SD and statistically analysed by Games-Howell’s or Tukey’s test. Asterisk (*) indicates significant difference (*p* < 0.05) in the viability compared to the negative control group

The vulnerability of PC-12 cells to high-dose corticosterone-induced toxicity was investigated through exposure to various concentrations of corticosterone ranged from 200 to 800 μM. As shown in Fig. 2a, cell viability was significantly decreased by all tested concentrations (*p* < 0.05). The viability gradually decreased with increasing corticosterone concentration from 200 to 500 μM, but was markedly reduced at 600 μM corticosterone. As 500 μM corticosterone produced more than 50% reduction in the viability (47.76 ± 4.93%; *p* < 0.05), 400 μM corticosterone was selected as the concentration for the induction of oxidative stress in the subsequent assays (65.77 ± 4.42%; *p* < 0.05).

### Effect of HESAE on the viability of PC-12 cells

The effect of HESAE on the viability of PC-12 cells was determined prior to the investigation of the neuroprotective activities of HESAE to exclude the possibility of cytotoxic and proliferative effects. The vulnerability of the cells to HESAE-induced toxicity was investigated through exposure to various concentrations of HESAE ranged from 0.125 to 8 mg/mL. As shown in Fig. 2b, there was no cytotoxicity observed for all tested concentrations. As the relatively lower concentrations of 0.25 to 1 mg/mL HESAE showed no significant difference in the viability compared to negative control (*p* > 0.05) and higher viability compared to 0.125, 2 and 4 mg/mL HESAE, the lower concentrations of HESAE (0.25 to 1 mg/mL) were selected to investigate its neuroprotective effects on viability as measured in the MTT and LDH leakage assays upon exposure to 400 μM corticosterone-induced cytotoxicity.

### Effect of HESAE on the viability of PC-12 cells treated with high-dose corticosterone

**Fig. 3:**
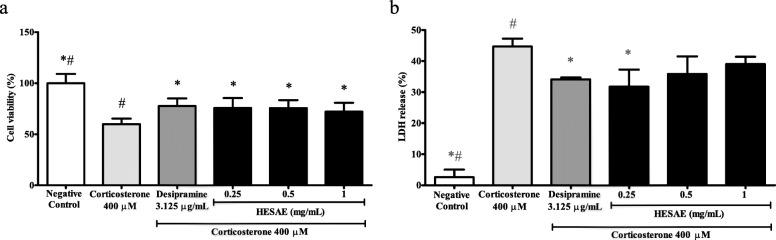
Effect of HESAE on the viability and LDH release in PC-12 cells treated with high-dose corticosterone. Cell viability and LDH release were evaluated by (**a**) MTT assay and (**b**) LDH assay following pre-treatment with HESAE for 48 h and high-dose corticosterone of 400 μM for 24 h. Data are expressed as mean ± SD and statistically analysed by Games-Howell’s or Tukey’s test. Asterisks (*) and hash signs (#) indicate significant differences (*p* < 0.05) in the viability and LDH release compared to the corticosterone group and desipramine group, respectively

The neuroprotective effects of HESAE against high-dose corticosterone-induced cytotoxicity was investigated through pre-treatment of HESAE at 0.25, 0.5 and 1 mg/mL for 48 h followed by 400 μM corticosterone for 24 h. As shown in Fig. 3a, 400 μM corticosterone significantly reduced the viability to 59.85 ± 5.49% or 1.7-fold lower compared to negative control (*p* < 0.05). However, pre-treatment with HESAE ranged from 0.25 to 1 mg/mL and desipramine significantly increased the viability to 75.66 ± 9.79% (0.25 mg/mL), 75.58 ± 7.70% (0.5 mg/mL), 72.19 ± 8.58% (1 mg/mL), and 77.68 ± 7.39% (desipramine) compared to corticosterone (*p* < 0.05). Pre-treatment with HESAE was observed to be comparable to the effect of desipramine in increasing the viability.

### Effect of HESAE on the LDH release in PC-12 cells treated with high-dose corticosterone

LDH is a soluble cytoplasmic enzyme that is released into extracellular space due to a damaged plasma membrane upon cell death. As shown in Fig. 3b, PC-12 cells treated with 400 μM corticosterone released 44.65 ± 2.60% LDH, being 17.1-fold higher compared to negative control (*p* < 0.05). However, pre-treatment with 0.25 mg/mL HESAE and desipramine for 48 h significantly reduced the LDH release to 25.63 ± 5.52% or 1.7-fold lower and 34.10 ± 0.60% or 1.3-fold lower compared to corticosterone, respectively (*p* < 0.05). There was no significant difference in the LDH release between higher concentrations of HESAE and corticosterone. Pre-treatment with 0.25 mg/mL HESAE was observed to be comparable to the effect of desipramine in reducing LDH release. As 0.25 mg/mL HESAE promoted highest viability as measured in the MTT and lowest LDH leakage, the concentration was selected for subsequent assays.

### Effect of HESAE on the SOD activity in PC-12 cells treated with high-dose corticosterone

**Fig. 4:**
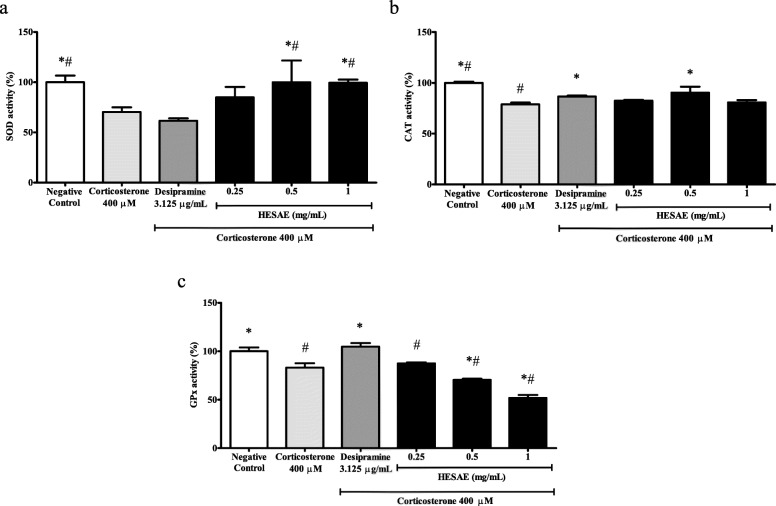
Effect of HESAE on endogenous antioxidant enzyme activities in PC-12 cells treated with high-dose corticosterone. **a** SOD, **b** CAT and **c** GPx activities were evaluated by SOD, CAT and GPx assays following pre-treatment with HESAE for 48 h and high-dose corticosterone of 400 μM for 24 h. Data are expressed as mean ± SD and statistically analysed by Tukey’s test. Asterisks (*) and hash signs (#) indicate significant differences (*p* < 0.05) in SOD, CAT and GPx activities compared to the corticosterone group and desipramine group, respectively

SOD is an endogenous antioxidant enzyme that catalyses the dismutation of superoxide anion (O_2_^−^) into hydrogen peroxide (H_2_O_2_) and oxygen (O_2_). As shown in Fig. 4a, PC-12 cells treated with 400 μM corticosterone significantly decreased the SOD activity to 70.24 ± 4.78% or 1.4-fold lower compared to negative control (*p* < 0.05). However, pre-treatment with 0.25 mg/mL HESAE did not increase the activity (*p* > 0.05). Neither 0.25 mg/mL HESAE nor desipramine increased the activity, and therefore higher concentrations of HESAE at 0.5 and 1 mg/mL were tested for SOD activity. Pre-treatment with 0.5 and 1 mg/mL HESAE significantly increased the activity up to 99.97 ± 21.6% and 99.46 ± 3.20% or 1.4-fold higher compared to corticosterone, respectively (*p* < 0.05) and 1.6-fold higher compared to desipramine (*p* < 0.05).

### Effect of HESAE on the CAT activity in PC-12 cells treated with high-dose corticosterone

CAT is an endogenous antioxidant enzyme that catalyses the dismutation of H_2_O_2_ into O_2_ and water (H_2_O). As shown in Fig. 4b, PC-12 cells treated with 400 μM corticosterone significantly decreased the CAT activity to 78.87 ± 1.72% or 1.3-fold lower compared to negative control (*p* < 0.05). However, pre-treatment with 0.25 mg/mL HESAE did not increase the activity (*p* > 0.05). Therefore, higher concentrations of HESAE at 0.5 and 1 mg/mL were tested for CAT activity. Pre-treatment with 0.5 mg/mL HESAE significantly increased the activity up to 90.46 ± 5.71% or 1.1-fold higher compared to corticosterone (*p* < 0.05) and this is comparable to the effect of desipramine in increasing CAT activity.

### Effect of HESAE on the GPx activity in PC-12 cells treated with high-dose corticosterone

GPx is an endogenous antioxidant enzyme that catalyses the dismutation of H_2_O_2_ into O_2_ and H_2_O. As shown in Fig. 4c, PC-12 cells treated with 400 μM corticosterone significantly reduced the GPx activity to 83.05 ± 4.63% or 1.2-fold lower compared to negative control (*p* < 0.05). However, pre-treatment with 0.25 mg/mL HESAE did not increase the activity (*p* > 0.05). Therefore, higher concentrations of HESAE at 0.5 and 1 mg/mL were tested for GPx activity. Nevertheless, the concentrations failed to restore the depleted activity. All tested concentrations of HESAE exhibited 1.2 to 2.0-fold lower in the activity compared to desipramine (*p* < 0.05).

### Effect of HESAE on the aconitase activity in PC-12 cells with high-dose corticosterone

**Fig. 5:**
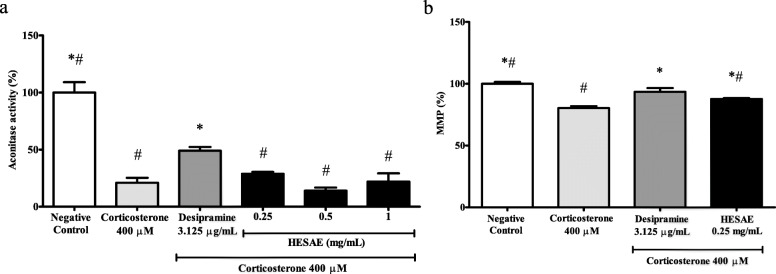
Effect of HESAE on aconitase activity and MMP in PC-12 cells treated with high-dose corticosterone. **a** Aconitase activity and **b** MMP were evaluated by aconitase and MMP assays following pre-treatment with HESAE for 48 h and high-dose corticosterone of 400 μM for 24 h. Data are expressed as mean ± SD and statistically analysed by Tukey’s test. Asterisks (*) and hash signs (#) indicate significant differences (*p* < 0.05) in aconitase activity and MMP compared to the corticosterone group and desipramine group, respectively

Aconitase is an iron-sulfur protein in the Krebs cycle that catalyses the isomerisation of citrate to isocitrate for energy production. As shown in Fig. 5a, PC-12 cells treated with 400 μM corticosterone significantly decreased the aconitase activity to 21.03 ± 4.24% or 4.8-fold lower compared to negative control (*p* < 0.05). However, pre-treatment with 0.25 mg/mL HESAE did not increase the activity (*p* > 0.05). Therefore, higher concentrations of HESAE at 0.5 and 1 mg/mL were tested for aconitase activity. Nevertheless, the concentrations failed to restore the depleted activity. All tested concentrations of HESAE exhibited 1.7 to 3.5-fold lower in the activity compared to desipramine (*p* < 0.05).

### Effect of HESAE on the MMP in PC-12 cells treated with high-dose corticosterone

The electrochemical membrane potential across the mitochondrial membrane or MMP is an essential component for energy storage during oxidative phosphorylation. JC-10 dye facilitates the discrimination of energised and de-energised mitochondria as indicated by red fluorescent aggregates that concentrate in the energised mitochondria as the membrane potential increases, whereas cells with collapsed MMP fail to retain the dye in the mitochondria resulting in the green fluorescence of its monomeric form. As shown in Fig. 5b, PC-12 cells treated with 400 μM corticosterone significantly decreased MMP levels to 80.31 ± 1.65% or 1.2-fold lower compared to negative control (*p* < 0.05). However, pre-treatment with 0.25 mg/mL HESAE significantly increased the MMP to 87.59 ± 0.78% or 1.1-fold higher compared to corticosterone (*p* < 0.05). The activity exhibited by HESAE was found to be 1.1-fold lower than that of desipramine (*p* < 0.05).

### Effect of HESAE on the intracellular ROS levels in PC-12 cells treated with high-dose corticosterone

**Fig. 6:**
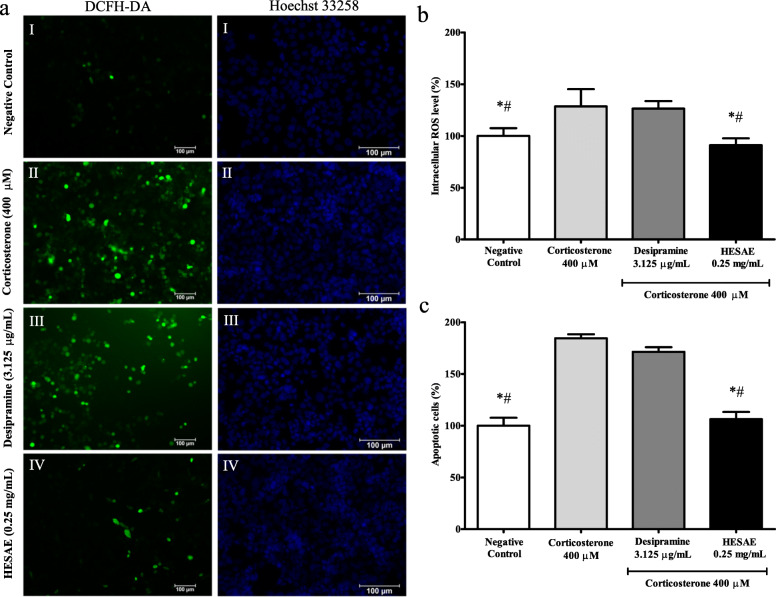
Effect of HESAE on the intracellular ROS levels and nuclear apoptosis in PC-12 cells treated with high-dose corticosterone. **a** DCFH-DA staining showing green fluorescence indicating intracellular ROS in PC-12 cells following pre-treatment with HESAE for 48 h and high-dose corticosterone of 400 μM for 24 h. Hoechst 33258 staining showing blue fluorescence indicating apoptotic nuclei in PC-12 cells following pre-treatment with HESAE for 48 h and high-dose corticosterone of 400 μM for 24 h. (I) Negative control; (II) cells treated with 400 μM corticosterone; (III) cells pre-treated with desipramine followed by exposure to high-dose corticosterone; and (IV) cells pre-treated with 0.25 mg/mL HESAE followed by exposure to high-dose corticosterone. Scale bar = 100 μm. **b** Effect of HESAE on intracellular ROS levels in high-dose corticosterone-treated PC-12 cells. Data are expressed as mean ± SD and statistically analysed by Tukey’s test. Asterisks (*) and hash signs (#) indicate significant differences (*p* < 0.05) in fluorescence intensity compared to the corticosterone group and desipramine group, respectively. **c** Effect of HESAE on percentage of apoptotic cells in high-dose corticosterone-treated PC-12 cells. Data are expressed as mean ± SD and statistically analysed by Tukey’s test. Asterisks (*) and hash signs (#) indicate significant differences (*p* < 0.05) in the percentage of apoptotic cells compared to the corticosterone group and desipramine group, respectively

Oxidation of DCFH-DA to DCF exhibits green fluorescence indicating the presence of ROS. As shown in Fig. 6a, PC-12 cells in the negative control group exhibited homogenously dim-green irregularly shaped structures after staining with DCFH-DA. 400 μM corticosterone caused an increase in green fluorescence compared to the negative control, indicating high intracellular ROS levels. However, pre-treatment with 0.25 mg/mL HESAE markedly decreased the fluorescence intensity. As shown in Fig. 6b, 400 μM corticosterone significantly increased the intracellular ROS levels from 100.00 ± 7.51% to 128.52 ± 16.74% (*p* < 0.05). Pre-treatment with 0.25 mg/mL HESAE significantly decreased the fluorescence intensity to 91.07 ± 6.62% or 1.4-fold lower compared to corticosterone (*p* < 0.05). The intensity induced by HESAE was also found to be 1.4-fold lower than that of desipramine (*p* < 0.05), demonstrating HESAE has higher capability in protecting the cells against damage by ROS compared to desipramine.

### Effect of HESAE on the nuclear apoptosis in PC-12 cells treated with high-dose corticosterone

Hoechst 33258 emits blue fluorescence upon binding with DNA to distinguish apoptotic cells from normal cells. As shown in Fig. 6a, PC-12 cells in the negative control group exhibited homogenously dim blue oval-shaped nuclei after staining with Hoechst 33258. 400 μM corticosterone caused an increase in bright blue fluorescence compared to the negative control, indicating cell apoptosis. However, pre-treatment with 0.25 mg/mL HESAE markedly decreased the fluorescence intensity. As shown in Fig. 6c, 400 μM corticosterone significantly increased the percentage of apoptotic cells from 100.00 ± 7.72% to 184.5 ± 3.85% (*p* < 0.05). Pre-treatment with 0.25 mg/mL HESAE significantly decreased the fluorescence intensity to 106.42 ± 6.84% or 1.7-fold lower compared to corticosterone (*p* < 0.05). The intensity induced by HESAE was found to be 1.6-fold lower than that of desipramine (*p* < 0.05), demonstrating HESAE has higher capability in protecting the cells against apoptosis compared to desipramine.

### Isolation and structural elucidation of major compounds of HESAE

**Fig. 7:**
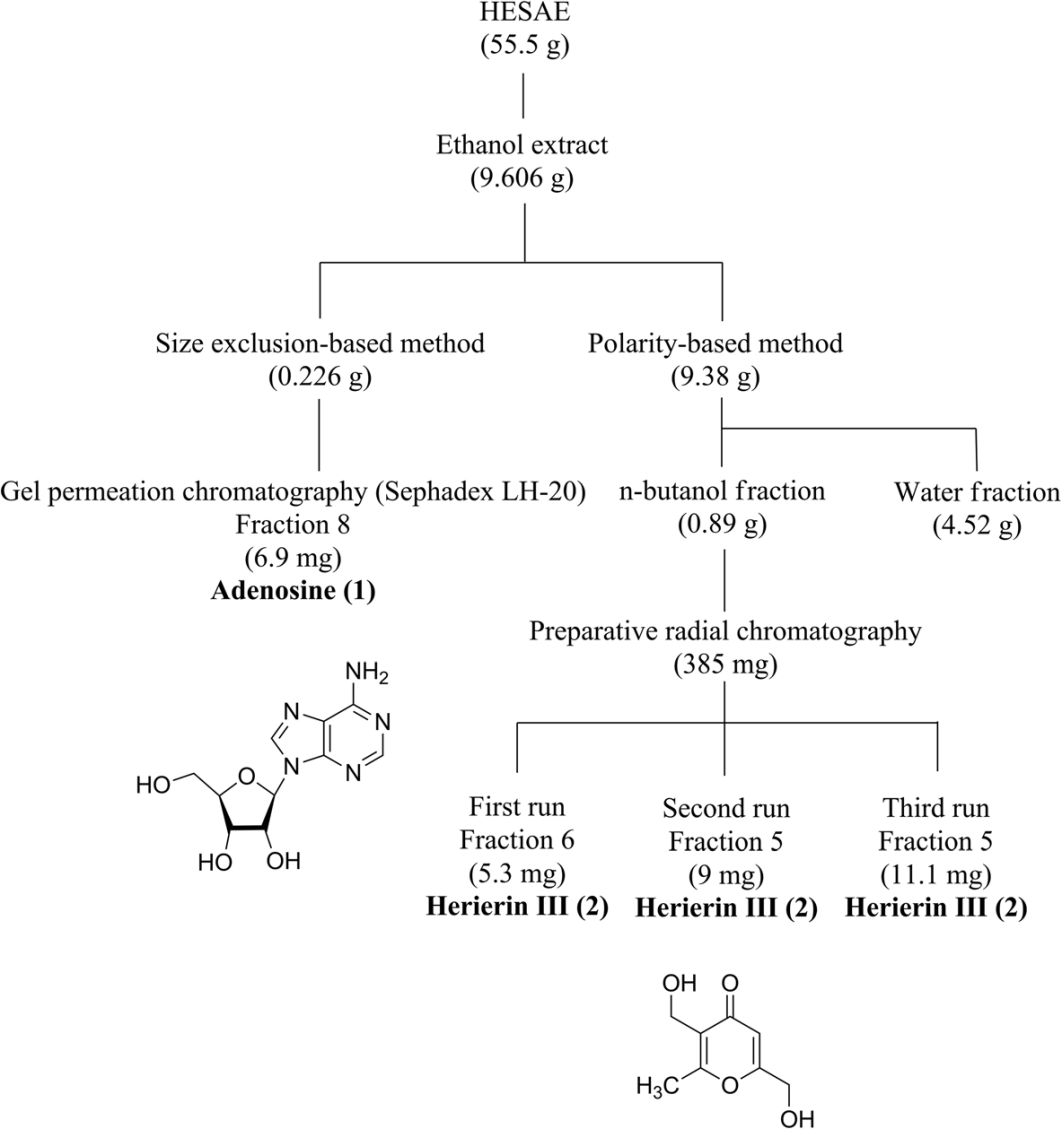
Isolation and structures of adenosine (1) and herierin III (2) from HESAE

Figure 7 shows the structures of two major compounds, namely adenosine (**1**) and herierin III (**2**) isolated from the ethanol extract of HESAE. Adenosine was isolated as a white powder. The UV spectrum showed absorption peak (λ_max_) at 207.5 and 259.5 nm, suggesting a typical adenine chromophore. In the IR spectrum, a broad peak was observed at 3362.71 cm^− 1^ due to the presence of OH groups. Absorption due to amine function was not observed due to overlapping with the observed OH peak in the IR spectrum. The HRESIMS showed an [M + H]^+^ peak at m/z 268.1043, which established the molecular formula of adenosine as C_10_H_13_N_5_O_4_.

Herierin III was isolated as a colorless oil. The UV spectrum showed absorption maxima at 213.0 and 251.0 nm, suggesting a pyrone chromophore. The IR absorption spectrum showed bands at 3325.42 cm^− 1^ and 1662.89 cm^− 1^ due to the presence of OH and unsaturated ketone functions. The molecular formula of herierin III was established as C_8_H_10_O_4_, based on HRESIMS spectrum where an [M + H]^+^ peak was observed at *m/z* 171.0653.

Structures of adenosine and herierin III were elucidated via the obtained NMR spectral data, including 1D (^1^H and ^13^C NMR) and 2D NMR [correlation spectroscopy (COSY), heteronuclear single quantum correlation (HSQC), heteronuclear multiple bond coherence (HMBC)]. Spectral data are in agreement with those reported in the literature for structural elucidation of adenosine [30] and herierin III [31]. The complete NMR data assignments of both compounds were summarised in Additional file 1.

Figure 7 shows a flow chart depicting the steps involved in isolation of the secondary metabolites. 95% ethanol was used as extracting solvent and the extraction yield was 9.606 g (17.31%). Size-exclusion chromatography using Sephadex LH-20 as the packing material and MeOH as the solvent system was employed to isolate polar compounds. Large molecules were eluted from the column first followed by progressively smaller molecules. The ^1^H NMR profiles of majority of the fractions showed overlapping of resonance signals due to sugars and secondary metabolites. Adenosine (6.9 mg, 0.46%) was isolated as a pure compound from the 8th fraction. In an attempt to separate the sugars from ethanol extract, the extract was subjected to pre-treatment with liquid-liquid partition between n-butanol and water. The yield of n-butanol and water fractions were 0.89 g (9.49%) and 4.52 g (48.19%), respectively. Non-polar compounds were extracted into n-butanol layer, while the sugars were extracted into water layer during the liquid-liquid partition process. n-butanol fraction was then fractionated using preparative radial chromatography with silica gel as stationary phase and CHCl_3_ as mobile phase with increasing MeOH gradient. The polarity-based method was suitable for the isolation of non-polar to medium polar compounds. Therefore, herierin III (25.4 mg, 0.05%), a medium polar compound was obtained from the 6th fraction of first run, and 5th fraction from second and third runs of preparative radial chromatography.

## Discussion

Phytochemicals and dietary antioxidants have been reported to protect cells against damage by ROS. Our previous findings showed that HESAE contains a substantial amount of phytochemicals [19]. HESAE was able to attenuate oxidative stress induced by L-Buthionine sulfoximine (BSO) in fibroblasts derived from a patient with Friedreich’s ataxia, which was attributed to the exogenous antioxidants of HESAE. Friedreich’s ataxia constitutes an excellent example of a pathology involving oxidative stress associated with excess iron uptake in cells [19]. Here, we extended our investigation of HESAE into its neuroprotective effects against oxidative stress in PC-12 cells that mimics the effects of depression. We are particularly interested in elucidating endogenous antioxidant defence capabilities of HESAE on the maintenance of mitochondrial function.

Antioxidants that inhibit the detrimental consequences of ROS are generally considered as promising therapeutic approach to neuroprotection [32]. An imbalance between the generation of ROS and the activity of antioxidant defence against ROS has been implicated in depression [33]. Oxidative stress caused by excessive ROS production has received considerable attention in recent years and has been proposed as a contributing factor to the pathophysiology of depression [34]. Furthermore, Behr et al. [35] highlighted the antioxidant effects of antidepressant drugs and the mechanisms of antioxidants executing the neuroprotective capabilities in the treatment of major depressive disorder (MDD).

Several cellular mechanisms of high-dose corticosterone-mediated oxidative stress in PC-12 cells have been documented including the generation of ROS [36], introduction of mitochondrial dysfunction [37] and fragmentation of DNA leading to apoptosis [38]. Consistent with this, we showed that high-dose corticosterone markedly reduced the viability of PC-12 cells and increased the release of LDH into the extracellular space, confirming neurotoxicity in PC-12 cells. However, pre-treatment with HESAE at 0.25 to 1 mg/mL prior to exposure to 400 μM corticosterone significantly increased the viability in a dose-dependent manner. Zhou et al. [39] and Yu et al. [40] observed a similar degree of apoptosis in PC-12 cells treated with 400 μM corticosterone, which support our findings on the dose of corticosterone that induces neurotoxicity in PC-12 cells.

Neuroprotective effects of HESAE might be mediated by non-enzymatic antioxidant defence systems involving phenolic compounds and flavonoids [19], and by enzymatic antioxidants including SOD, CAT, GPx, glutathione reductase (GR) and paraoxonase 1 (PON1). In this study, pre-treatment with HESAE successfully restored the SOD and CAT activities confirming the enzymatic antioxidant defence mechanisms of HESAE. Our findings are in accordance with Wang et al. [41] and Han et al. [42]. Wang et al. [41] found that a polysaccharide derived from the mycelium (designated as EP-1) increased intestinal serum SOD activity in a rat model of acetic acid-induced ulcerative colitis and in human epithelial colorectal adenocarcinoma cellular model of H_2_O_2_-induced oxidative stress. Han et al. [42] found that a polysaccharide derived from the fruiting bodies increased CAT levels in a rat model of renal ischemia-reperfusion injury. The SOD is important in controlling cellular ROS levels, as it is responsible for the dismutation of O_2_^−^, the principal ROS formed by the mitochondria to H_2_O_2_ and O_2_.

On the other hand, pre-treatment with HESAE failed to restore the activity of GPx following exposure to high-dose corticosterone. We hypothesised that the neuroprotective effects of HESAE were mediated through a glutathione-independent mechanism which resulted in compensatory increased in SOD and CAT. CAT serves as a major defence in high level of oxidative stress especially when there is a limited content of glutathione or decreased GPx activity, whereas glutathione redox cycle involving GPx serves as a major defence in low level of oxidative stress [43].

Depression is a complex multifactorial disease with many possible causes. Major depressive disorder is associated with mitochondrial dysfunction leading to oxidative stress [44]. Mitochondria are widely recognised as a source of ROS, and overproduction of ROS leads to compromised antioxidant defence systems and oxidative stress. The interaction between energy metabolism and ROS is evident during the aging process and in the onset and progression of many age-related diseases. Upon exposure to high-dose corticosterone, dysfunction in mitochondrial autophagy machinery and impaired autophagy may increase mitochondrial ROS formation [45]. In this study, we observed that PC-12 cells treated with high-dose corticosterone had decreased cell viability, reduced endogenous antioxidant enzyme activities, disrupted mitochondrial function, and increased oxidative stress and apoptosis. Pre-treatment of PC-12 cells with HESAE prior to the oxidative stress had prevented mitochondrial dysfunction, reduced intracellular ROS levels and attenuated apoptosis.

Metabolic enzymes, such as α-ketoglutarate dehydrogenase and aconitase, are highly sensitive to O_2_^−^. Aconitase has been found in both cytosol and mitochondria, and it is involved in the metabolic regulation of iron and as a Krebs cycle intermediate for maintaining ROS homeostasis [46]. Nevertheless, our findings reveal that pre-treatment with HESAE at higher concentrations of 0.5 and 1 mg/mL did not appear to increase the aconitase activity upon exposure to high-dose corticosterone. This is in accordance with Lian and Stringer [47] who demonstrated that a pre-treatment with glutamine, an exogenous antioxidant did not prevent the decrease in aconitase activity in the rat cortical astrocytes after exposure to cortical spreading depression and fluorocitrate, a selective inhibitor of astrocytic Krebs cycle.

Monitoring the relative MMP in living cells can be a direct measurement of mitochondrial function. The mitochondrial electron transport chain creates an electrochemical gradient of protons across the mitochondrial inner membrane driving ATP synthesis via mitochondrial ATP synthase [48]. The electron transport chain is also involved in the formation of O_2_^−^ [49]. Excessive ROS production induces the rapid depolarisation of the mitochondrial inner membrane potential and subsequently impairs oxidative phosphorylation. Disruption of MMP triggers the opening of mitochondrial permeability transition pore (mPTP) and the release of pro-apoptotic proteins leading to cell apoptosis [50]. In this study, high-dose corticosterone reduced MMP levels in PC-12 cells, which is in line with previous findings [39, 51]. Conversely, pre-treatment of PC-12 cells with HESAE restored MMP levels, a key indicator of mitochondrial activity.

Excess cellular ROS levels can activate apoptotic cell death pathways. Morphological changes associated with apoptosis include cell shrinkage, chromatin condensation and formation of apoptotic bodies, leading to changes in plasma membrane permeability and plasma membrane damage [52]. In this study, HESAE was shown to attenuate apoptosis following exposure to high-dose corticosterone. Likewise, a biomass preparation of *H. erinaceus* also showed protective activity against Di-2-ethylhexyl-phthalate (DEHP)-induced apoptosis mediated by caspase activation [53].

HESAE was further extracted with ethanol to obtain compounds with lower polarity and the phytochemical analysis revealed the presence of adenosine and herierin III. Hydro-alcoholic extractions have been recommended for the recovering of biologically active compounds of mushrooms including phenolic compounds and flavonoids [54, 55]. Further, Jiang et al. [54] showed that anhydrous ethanol appeared to be the most efficient solvent in the extraction of high-polarity components of *H. erinaceus* contributing to high antioxidant activity as measured by reducing power and free radical scavenging assays.

Adenosine from *H. erinaceus* fruiting bodies was first discovered by Mizuno [5]. It is an endogenous nucleoside classified under glycosylamines consisting of an adenine attached to a ribose [56]. The yield of adenosine at 0.46% is 3.3-fold higher compared to that of *Ganoderma lucidum* (Curtis) P. Karst. (0.14%) [57] but almost 2-fold lower than that of *Cordyceps militaris* (L.) Fr. (0.81%) [58]. Adenosine is a neuromodulator that acts through multiple mechanisms, including regulation of neurotransmitter releases from synaptic vesicles, neuronal hyperpolarisation or depolarisation, and glial cell activity [59]. Adenosine has both excitatory and inhibitory effects through its A_1_ and A_2A_ receptors [60]. On the other hand, isolation of herierin III from *H. erinaceus* mycelium grown on solid media was pioneered by Qian et al. [61] followed by its discovery in the mycelium produced by submerged fermentation [62]. Herierin III is a heterocyclic compound classified under pyrones. The yield of herierin III at 0.05% is 5-fold higher than the amount (0.01%) reported by Zhang et al. [62]. Although the compound has also been isolated from mycelium of *Morchella* Dill. ex Pers. [31], *Hericium americanum* Ginns*, Hericium abietis* (Weir ex Hubert) K.A. Harrison and *Hericium alpestre* Pers. [63], its beneficial effects in human health is still poorly understood. Taken together, further investigation on the neuroprotective effects of adenosine and herierin III derived from *H. erinaceus* can be conducted in pre-clinical models of depression.

**Fig. 8:**
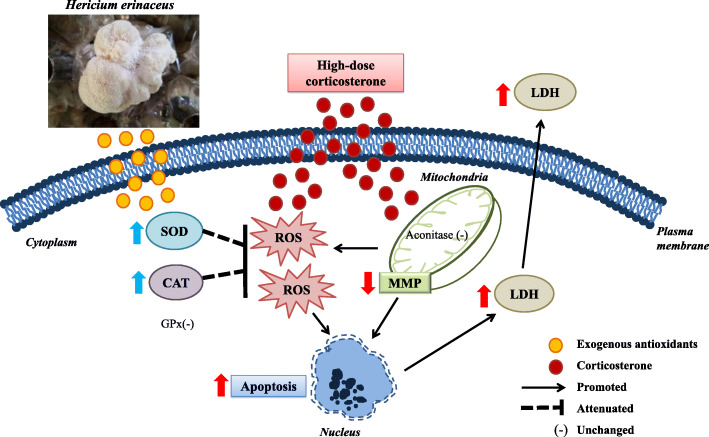
Proposed neuroprotective effects of HESAE against high-dose corticosterone-induced oxidative stress in PC-12 cells. The illustrations were prepared electronically using Microsoft PowerPoint 2016. The image of *H. erinaceus* was captured at mushroom cultivation farm of Ganofarm R&D Private Limited with permission from Ganofarm R&D Private Limited

The possible neuroprotective mechanisms of HESAE are presented in Fig. 8. We propose that the exogenous antioxidant compounds in HESAE possess neuroprotective effects against high-dose corticosterone-induced oxidative stress in PC-12 cells. The antioxidants can pass through the plasma membrane into the cytoplasm and mitochondria. They upregulated endogenous antioxidant enzyme activities of SOD and CAT, prevented mitochondrial dysfunction as evidenced by the restoration of MMP levels and the prevention of MMP depolarisation in the inner mitochondrial membrane, reduced intracellular ROS levels, decreased LDH release and inhibited apoptosis. Reconstruction of mitochondrial functions showed the prevention of downstream apoptotic cascades in the maintenance of cellular integrity. However, these antioxidants failed to increase the depleted levels of GPx in the cytoplasm and aconitase in the mitochondrial matrix. Remarkably, the neuroprotective effects were similar to that of desipramine, a secondary amine tricyclic antidepressant and a potent inhibitor of the noradrenaline reuptake. However, the anti-cholinergic and H_1_-antihistaminergic activities of desipramine can cause adverse effects such as blurred vision, postural hypotension, dry mouth, tremors, seizures, confusion, delirium and sedation [64].

## Conclusions

Our study demonstrated the neuroprotective effects of HESAE via upregulation of antioxidant enzyme activities in an in vitro model of depression. The neuroprotective effects could possibly be attributed to the major compounds found in HESAE, namely adenosine and herierin III. HESAE could be a potential dietary supplement to be used to treat depression. Further pre-clinical trials are warranted to validate the efficacy and safety of adenosine and herierin III as mitochondria-targeted antioxidants.

## Supplementary Information

**Additional file 1. **NMR data assignments. **Table S1.** ^1^H and ^13^C NMR Spectroscopic Data of Adenosine (1). **Table S2.** ^1^H and ^13^C NMR Spectroscopic Data of Herierin III (2).

## Data Availability

The datasets used and/or analysed during the current study are available from the corresponding author on reasonable request.

## Abbreviations

HESAE: *Hericium erinaceus* standardised aqueous extract
PC-12: Pheochromocytoma cells 12
LDH: Lactate dehydrogenase
MMP: Mitochondrial membrane potential
ROS: Reactive oxygen species
HPA: Hypothalamic-pituitary-adrenal
MTT: 3-(4,5-dimethylthiazol-2-yl)-2,5-diphenyltetrazolium bromide
SOD: Superoxide dismutase
CAT: Catalase
GPx: Glutathione peroxidase
DCFH-DA: 2′,7′-dichlorofluorescin diacetate
MeOH: Methanol
CHCl_3_: Chloroform
NMR: Nuclear magnetic resonance
HRESIMS: High-resolution electrospray ionisation mass spectrometry
UV: Ultraviolet
IR: Infrared
COSY: Correlation spectroscopy
HSQC: Heteronuclear single quantum correlation
HMBC: Heteronuclear multiple bond coherence
