# Supplementary material for: Neuroprotective effects of Hericium erinaceus (Bull.: Fr.) Pers. against high-dose corticosterone-induced oxidative stress in PC-12 cells
Source: BMC Complement Med Ther. 2020 Nov 11;20:340. doi: 10.1186/s12906-020-03132-x (PMC7656699; doi:10.1186/s12906-020-03132-x)
Supplement: Supplementary file 1 — Additional file 1. NMR data assignments. Table S1. 1H and 13C NMR Spectroscopic Data of Adenosine (1). Table S2. 1H and 13C NMR Spectroscopic Data of Herierin III (2). [file 12906_2020_3132_MOESM1_ESM.docx]

**Additional file 1 -** NMR data assignments





**Table 1.** ^1^H and ^13^C NMR Spectroscopic Data of Adenosine (**1**).

| **Position** | **^1^H (ppm)^1^** | **^13^C (ppm)^2^** |
| --- | --- | --- |
| 2 | 8.32, s | 153.3 |
| 4 |  | 150.0 |
| 5 |  | 120.0 |
| 6 |  | 168.7 |
| 8 | 8.19, s | 142.0 |
| 1’ | 5.97, d (6.4) | 91.2 |
| 2’ | 4.43, br s | 75.5 |
| 3’ | 4.40, br s | 72.6 |
| 4’ | 4.21, br s | 88.2 |
| 5’ | 3.80, m | 63.4 |
| NH_2_ | 6.62, br s |  |

Assignments based on COSY, HSQC and HMBC. Recorded at 600 MHz in MeOH-*d*_4_.^1^ Recorded at 150 MHz in MeOH-*d*_4_.^2^

**Table 2.** ^1^H and ^13^C NMR Spectroscopic Data of Herierin III (**2**).

| **Position** | **^1^H (ppm)^1^** | **^13^C (ppm)^2^** |
| --- | --- | --- |
| 2 |  | 167.5 |
| 3 | 6.40 s | 111.4 |
| 4 |  | 180.8 |
| 5 |  | 123.7 |
| 6 |  | 163.2 |
| 7 | 4.46 s | 61.0 |
| 8 | 4.52 s | 57.3 |
| 6-CH_3_ | 2.37 s | 17.2 |

Assignments based on COSY, HSQC and HMBC. Recorded at 600 MHz in MeOH-*d*_4_.^1^ Recorded at 150 MHz in MeOH-d_4_.^2^
